# Chemical and Structural Changes in Corn Stover After Ensiling: Influence on Bioconversion

**DOI:** 10.3389/fbioe.2020.00739

**Published:** 2020-08-14

**Authors:** Nick J. Nagle, Bryon S. Donohoe, Edward J. Wolfrum, Erik M. Kuhn, Thomas J. Haas, Allison E. Ray, Lynn M. Wendt, Mark E. Delwiche, Noah D. Weiss, Corey Radtke

**Affiliations:** ^1^National Renewable Energy Laboratory, Golden, CO, United States; ^2^Idaho National Laboratory, Idaho Falls, ID, United States; ^3^Department of Chemical Engineering, Lund University, Lund, Sweden; ^4^Verd Company, Houston, TX, United States

**Keywords:** feedstock logistics, ensiling, bioconversion, pretreatment, ethanol, bioproducts

## Abstract

Production of biofuels, bioproducts, and bioenergy requires a well-characterized, stable, and reasonably uniform biomass supply and well-established supply chains for shipping biomass from farm fields to biorefineries, while achieving year-round production targets. Preserving and stabilizing biomass feedstock during storage is a necessity for cost-effective and sustainable biofuel production. Ensiling is a common storage method used to preserve and even improve forage quality; however, the impact of ensiling on biomass physical and chemical properties that influence bioconversion processes has been variable. Our objective in this work was to determine the effects of ensiling on lignocellulosic feedstock physicochemical properties and how that influences bioconversion requirements. We observed statistically significant decreases (*p* < 0.05) in the content of two major structural carbohydrates (glucan and xylan) of 5 and 8%, respectively, between the ensiled and non-ensiled materials. We were unable to detect differences in sugar yields from structural carbohydrates after pretreatment and enzymatic hydrolysis of the ensiled materials compared to non-ensiled controls. Based on this work, we conclude that ensiling the corn stover did not change the bioconversion requirements compared to the control samples and incurred losses of structural carbohydrates. At the light microscopy level, ensiled corn stover exhibited little structural change or relocation of cell wall components as detected by immunocytochemistry. However, more subtle structural changes were revealed by electron microscopy, as ensiled cell walls exhibit ultrastructural characteristics such as wall delimitation intermediate between non-ensiled and dilute-acid-pretreated cell walls. These findings suggest that alternative methods of conversion, such as deacetylation and mechanical refining, could take advantage of lamellar defects and may be more effective than dilute acid or hot water pretreatment for biomass conversion of ensiled materials.

## Introduction

The United States Department of Energy’s *2016 Billion-Ton Report: Advancing Domestic Resources for a Thriving Bioeconomy* (Langholtz et al., 2016) projects that 1.0 billion tons of biomass will be available by 2030 and 1.2 billion tons by 2040. Conversion of the 1.2 billion metric tons of biomass could result in the production of 50 billion gallons of biofuels, 50 billion pounds of bio-based chemicals and bioproducts, and 85 billion kilowatt-hours of electricity to power 7 million households (Rogers et al., 2016). This resource would contribute 1.1 million jobs to the U.S. economy and keep $260 billion in the United States. Additionally, the collection, conversion, and utilization of the 2030 biomass volume targets “could displace 9.5% of fossil energy consumption and avoid as much as 446 million tons of CO_2_ equivalent emissions annually” (Rogers et al., 2016). Secondary impacts include bolstering rural economies, creating jobs, and improving both soil and water quality through application of advanced agronomic practices (U.S. Department of Energy Office of Energy Efficiency and Renewable Energy, 2015; Bonner et al., 2016). Achieving the needed economics and logistics required not only improved biomass conversion processes, but more importantly, biomass supply chains that reduce risk and allow for commodity processing of agricultural residues, energy crops, and lower-value waste feedstocks. Some of the largest barriers for cellulosic-based fuels and chemicals do not reside so much in the biorefinery as in the upstream operations of biomass harvesting, handling, storage, transport, and pretreatment/preprocessing (Dale, 2017; Rivers, 2018). Biomass storage is one of these critical upstream challenges (Darr and Shah, 2012; Ebadian et al., 2017). Inman et al. (2010), Rogers et al. (2016) reported that in order to achieve the 2022 Renewable Fuel Standard (RSF) production target of 21 billion gallons of biofuel, over 254 billion metric tons of biomass would be required. The majority of the tonnage would have to be stored and stabilized to provide a consistent year-round supply. The inventory level needed to achieve this degree of consistent, stable biomass supply would require a storage area of 1.37 billion m^3^, which is over twice the 0.62 billion m^3^ available for grain storage in 2014 (Darr and Shah, 2012). While grain bins and other well-established agricultural infrastructures have been used to reduce grain losses by minimizing dry-matter loss and exposure to moisture (Darr and Shah, 2012; Smith et al., 2013; Wendt et al., 2018b), the fibrous nature and low density of lignocellulosic biomass—primarily agricultural residues—increase the challenges for biomass storage and transport compared to grain (Dale, 2017). Currently, different variations of dry bale storage systems exist, including different ground covers, top covers, and storage shelters. Regardless, the most common practice is to aggregate biomass prior to delivery to the biorefinery. These practices have shown dry-matter losses (DML) of <10%, increasing with increased moisture content, and have been previously described in some detail (Darr and Shah, 2012; U.S. Department of Energy Office of Energy Efficiency and Renewable Energy Bioenergy Technologies Office, 2016; Dale, 2017; Wendt et al., 2018a, b). Drying time prior to baling varies seasonally, and incomplete drying can lead to biological degradation and self-heating, increasing DML and the risk of spontaneous combustion (Smith et al., 2013; Wendt et al., 2018a; Webb and Chambers, 2019). Fires in stacked bale storage systems lead to major losses in stored biomass, have proven to be difficult to control, and potentially create health risks from smoke and small particle inhalation. Fires in biomass stack yards have occurred in several of the large cellulosic ethanol plants (U.S. Department of Energy Office of Energy Efficiency and Renewable Energy Bioenergy Technologies Office, 2016; Webb et al., 2018). Reducing fire risk has focused on alternative bale stacking methods and providing more space between stacks to reduce fire size and spread, but fire risk has not been eliminated (U.S. Department of Energy Office of Energy Efficiency and Renewable Energy Bioenergy Technologies Office, 2016).

An alternative to dry storage in silos or stacked bale yards is a wet storage system, also known as ensiling, has been employed to preserve feedstock for livestock forage. Ensiling is achieved through fermentation by anaerobic bacteria, primarily heterofermentative and homofermentative lactic acid strains. These microbes ferment free sugars, lower the pH by producing carboxylic acids such as acetic, butyric, propionic, and lactic acids, while further reducing the oxygen content, thus creating an anaerobic environment (Chen et al., 2007; Smith et al., 2013; Essien et al., 2018). These final low-pH and low-O_2_ conditions reduce microbial activity and preserve biomass.

The potential benefits of ensiling in the context of a biorefinery supply system include reduced dependence on seasonality for biomass harvesting thus allowing for a wider harvest window; reduced DML; and reduced fire risk. Ensiling may provide additional benefits by reducing handling and preprocessing challenges, such as size reduction prior to ensiling, negating the need for additional preprocessing at the biorefinery (Wendt et al., 2018b). Reduction in pretreatment severity required for bioconversion of ensiled biomass has been reported by some groups (Essien et al., 2018), but the overall results in the bioconversion between field and lab studies have been variable (Wendt et al., 2018a). Variables that can influence the impact of ensiling on bioconversion include the type and variety of the feedstock; ensiling methodology and use of additives; chemical treatments such as alkaline, dilute acid, or wet oxidation; type of bioconversion process and/or enzymatic hydrolysis; and, finally, the scale of the studies (Wendt et al., 2018a).

Techno-economic analysis using corn stover, comparing a field chopped logistics system incorporating ensiling to a bale-based logistics system, reported that cost per dry ton for the chopped logistics system was slightly higher compared to the bale logistics system: $137.86 and $125.70, respectively (Wendt et al., 2018b). Additional benefits such as enhanced fermentation of ensiled materials for carboxylic acid fermentation (Lin et al., 2016; Nelson et al., 2017) may increase production amounts for both fuels and chemicals. Achieving reduction of pretreatment requirements resulting from wet storage may be more challenging; inconsistent results for wet storage when accounting for differences in variation in the feedstock, harvesting and collection practices, and storage regimes overshadow smaller improvements in reducing pretreatment requirements when operating at larger production scales.

Results from smaller-scale studies (Chen et al., 2007) have evaluated the enzymatic hydrolysis of materials ensiled both with and without enzyme addition, comparing the sugar release and holocellulose (cellulose and hemicellulose) losses after enzymatically hydrolyzing untreated and ensiled feedstocks. Ensiling significantly (*p* < 0.05) increased the sugar release and holocellulose loss in cotton stalks, wheat straw, and barley compared to untreated feedstocks. However, Chen et al. were unable to detect changes in sugar release or holocellulose loss upon adding enzymes at the start of the ensiling process (*p* < 0.05). In a companion study (Chen et al., 2007), these researchers compared chemically pretreated feedstocks to enzyme-assisted ensiled feedstocks. The sugar and ethanol yields from the pretreated feedstocks were higher than yields from the enzyme-assisted ensiled wheat straw and triticale; yields from the enzyme-assisted ensiled hay materials were comparable to yields from the chemically treated materials.

Wet oxidation has been successfully used to pretreat whole-crop maize (Thomsen et al., 2008; Xu et al., 2010; Essien et al., 2018). The combined pretreatment and enzymatic hydrolysis of the ensiled green maize, described previously, resulted in up to >90% and >80% yields from glucan and xylan hydrolysis, respectively, depending on pretreatment condition. However, these studies were not compared to a non-ensiled sample to understand the potential change in recalcitrance reduction that occurred as a result of storage. Simultaneous saccharification and fermentation (SSF) of the pretreated ensiled maize residues showed no inhibition and, in several cases, achieved 95% to 98% of theoretical ethanol yields (Thomsen et al., 2008). Oleskowicz-Popiel et al. (2011) reported that ethanol yields from SSF were highest in all three of the ensiled and hydrothermally treated maize, rye, and clover biomass, reaching 80% of theoretical yield when compared to ethanol yields from the conversion of maize, rye, clover, and non-ensiled biomass using hydrothermal pretreatment. Anaerobic digestion of ensiled herbaceous feedstocks such as grasses and corn stover has shown increased biogas production (Janke et al., 2019), with 71% of methane potential achieved from ensiled sugar cane combined with molasses, an ensiling additive. However, no increase in methane potential was observed when materials were ensiled without additives.

Previous studies have explored some of the range of variables highlighted above, and most were performed at a smaller scale (Wendt et al., 2018a), making comparisons to larger-scale performance difficult. The specific objectives of the work presented here were to (1) compare the effects of two pretreatment processes on the digestibility of ensiled and non-ensiled corn stover, with and without ensiling additives; (2) characterize the yields from both conversion processes using compositional analysis of both materials; and (3) examine the microscale structure of both the ensiled and non-ensiled corn stover to determine the level of structural changes occurring from ensiling to gain a better understanding of mechanisms of preservation and conversion.

## Materials and Methods

### Corn Stover Ensiling

**FIGURE 1:**
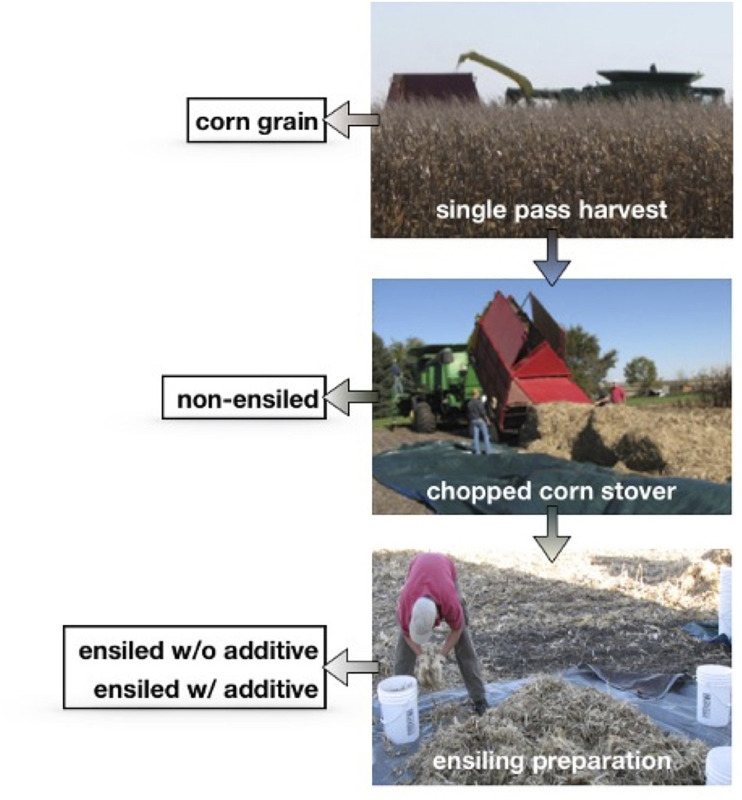
Schematic diagram of the sampling scheme used in this work. All samples were taken from a single tarp containing approximately 50 kg of chopped corn stover collected with a single-pass harvester. Ensiling was set up with and without additives the same day as the harvest. Samples of the stover from the tarp were frozen and dried in a 45°C oven, also on the same day as the harvest.

Corn stover was collected from study plots at the Iowa State University Agronomy and Agricultural Engineering Farm in Ames, Iowa. The harvesting and ensiling setup was conducted using the whole (100% removal) cut fraction. The corn stover and grain were harvested at the time of grain maturity at approximately 49% total solids (w/w). A single-pass harvester simultaneously harvested the corn grain and separated it from the stover. The chopped stover (approximately 52 kg) was blown into a wagon pulled behind the harvester. The wagon was then unloaded onto a 30-ft by 50-ft tarp (Figure 1). Randomized representative subsamples from the stover were collected and either stored at −20°C or dried at 45°C and then stored at ambient temperature. Approximately 5 kg (10%–12%) of the collected stover was either frozen or field dried, leaving approximately 47 kg of stover available for ensiling.

The remaining corn stover was divided into two groups: one was ensiled with additives, the other without. The materials ensiled with additives were prepared using Silamax 50 WS (from Chemorse in Des Moines, Iowa), which contains *Enterococcus faecium*, *Lactobacillus plantarum*, and *Pediococcus acidilactici* bacteria. The additive also contains cellulase, fungal and bacterial amylases, proteases, and beta-glucanase enzymes. A subsample of corn stover was taken from the initial stover pile and mixed, using shovels, with Silamax 50 WS, representing an application rate of 1.75 kg/t fresh stover. One liter of water was sprayed on the pile during mixing to wet the additive. The ensiling reactors consisted of 19-liter buckets with gas seals attached to the lids, *n* = 3 for corn stover ensiled with and without additives. The reactors were filled to the top with either untreated or additive-treated corn stover, at a packing density of approximately 74.4 kg Dry Material (DM)/m^3^. The buckets with the ensiled corn stover were stored in an environmental chamber at 37°C to simulate the center of a silage pile.

Prior to analysis, samples (frozen, dried, and ensiled with and without additives) were subsampled using the Pierre Gy method (Pitard, 1993) and reduced in size to 6.25 mm using a Wiley knife mill (from Thomas Scientific in Swedesboro, NJ, United States). Subsamples of the material were further milled to 2 mm and used for all compositional analysis. All samples were oven dried at 60°C prior to compositional analysis. A schematic diagram of samples showing both the collection and ensiling of the corn stover is shown in Figure 1.

### Corn Stover Analysis

The chemical composition for all dried, frozen, and ensiled feedstock samples was determined using the National Renewable Energy Laboratory (NREL’s) standard laboratory analytical procedures (LAPs) (National Renewable Energy Laboratory [NREL], 2013). Briefly, biomass is first extracted sequentially with water and ethanol using an Accelerated Solvent Extractor (ASE Dionex CA, model 200). Solvent was removed using a rotary evaporator, then dried and weighed. Structural carbohydrates were determined using a two-step acid hydrolysis to fractionate the biomass into forms that are more easily quantified. The extracted biomass was digested using 72% (w/w) sulfuric acid, then diluted to 4% sulfuric acid and further digested at 121°C for 60 min. Samples were neutralized and filtered prior to analysis using Agilent Technologies (Santa Clara, CA, United States) model 1100 HPLC and the BioRad Aminex HPX-87P column (Hercules, CA, United States). Acid-insoluble lignin is determined by rinsing the filtered solids with deionized water, followed by drying and then weighing to determine the amount of remaining lignin.

Starch was determined from the Association of Official Agricultural Chemists [AOAC] (1998) (Megazyme, 2014). Extracted and non-extracted biomass was analyzed for starch using a two-enzyme digestion of the solid biomass. A 100-mg sample was first treated with 190-proof ethanol, followed by an addition of dimethyl sulfoxide, and then immersed in boiling water for 5 min. Three hundred units of α-amylase (from Megazyme in Wicklow, Ireland) were then added and incubated in boiling water for 6 min. The samples were then incubated in a 50°C water bath, followed by the addition of 0.1 mL (20 units) amyloglucosidase (from Megazyme), and incubated for 30 min at 50°C. Samples were centrifuged, filtered, and analyzed for glucose by high-performance liquid chromatography (HPLC).

The pretreated samples were analyzed, focusing on the liquor for monomeric and oligomeric sugars, acetate, lignin, furfural, and hydroxymethylfurfural (HMF) using the NREL LAPs (National Renewable Energy Laboratory [NREL], 2013). Percentage of total solids (%) was calculated on the whole pretreated slurry to determine the mass of remaining solids. The remaining cellulose was calculated by subtracting the mass of the solubilized glucose in the liquor phase from the initial cellulose content in the sample. The fraction of remaining cellulose was then multiplied by the mass of remaining solids to determine the mass of cellulose in the solids. This number was used to determine the cellulase loading.

### Organic Acid Analysis

Samples were homogenized and aqueous analytes were extracted from the solids using a modification of the method described in Carr et al. (1984). Solids were ground (1:10 w/v) for 60 s in a laboratory blender (Waring model 3390D25) and filtered through a syringe filter (Whatman GD/X; 0.2 μm, non-sterile). Organic acids were analyzed by HPLC using a Bio-Rad Aminex HPX-87H column.

### Pretreatment Experiments

All corn stover samples were pretreated using a MultiClave 10X Reactor (Autoclave Engineers in Erie, PA, United States): 170°C reaction temperature, 6.5-min residence time, 0.07 g/g H_2_SO_4_ (wt. acid/wt. biomass); and 200°C reaction temperature, 20-min residence time, no acid addition. A 5% solids loading was used for all samples, resulting in a total mass per well of 25 g. Each pretreatment condition was done in triplicate. Thus, a total of 48 experiment samples were generated: 8 samples (3 ensiled samples with additives, 3 ensiled samples without additives, 1 field-dried sample, and 1 frozen sample), each pretreated under two conditions (dilute sulfuric acid and water-only) with three replicates. A control corn stover material (Pioneer 33A14 variety) was included in one of the pretreatment wells, totaling six additional samples, to serve as a method validation standard for the pretreatment.

The MultiClave reactor was heated using two sand baths (Techne Inc., Cambridge, United Kingdom) for the pretreatment experiments. The larger industrial bath (Model IFB-121) was set at 230°C to accelerate the heat-up period, and the smaller sand bath was set at the desired reaction temperature. When the internal reactor temperature was within 10°C of the target temperature, the reactor was transferred from the larger sand bath into the smaller sand bath to maintain reaction temperature for the duration of the experiment. Temperature was monitored throughout the experiment. Immersing the reactor into a bucket of ice quenched the reaction.

Pretreated slurries were removed from the reactor and separated into solid and liquid fractions using a vacuum flask and glass-fiber filters. The liquors were analyzed for monomeric and total sugars, organic acids, and solids content. The pretreated solids were washed with deionized water, and the solids content and total weight were recorded. These data were used to determine the amount of sugars hydrolyzed during pretreatment as well as the cellulose content of the pretreated biomass required for enzyme loading.

### Enzymatic Hydrolysis Experiments

All corn stover samples (ensiled and non-ensiled) were enzymatically hydrolyzed using a modified protocol by Dowe (2009). A commercial cellulase (GC220 from Genencor in Rochester, NY, United States), was loaded at 40 mg protein per gram cellulose (24 Filter Paper Units (FPU)per gram cellulose) with a beta-glucosidase (β-G)activity of 232 μmol/min/mL. The Genencor enzyme loading was based on the residual cellulose remaining in the pretreated material. Samples were incubated for 5 days at 48°C on a shaking incubator set to 100 rpm. The liquor from the post-enzymatic hydrolysis slurry was separated using a syringe filter (0.45 μm) and analyzed for monomeric sugars, based on NREL LAPs (National Renewable Energy Laboratory [NREL], 2013).

### Yield Calculations

The structural carbohydrate yield from cellulosic materials is a critical measure of the pretreatment and enzymatic hydrolysis performance. It is defined as the fraction of a given carbohydrate mass recovered from the aqueous biomass slurry in either monomeric or oligomeric form. This occurs after either pretreatment alone or after a sequential pretreatment and enzymatic hydrolysis. In this work, we calculated both the glucan and xylan yield for both the dilute acid and the hot water pretreatment processes along with enzymatic hydrolysis of the pretreated solids. We did not include degradation products from pretreatment, such as furfural and hydroxy-methyl furfural, in these yield calculations. The combined sugar release from both pretreatment and enzymatic hydrolysis, weighted by their initial mass fraction, is defined as feedstock total carbohydrate yield and is calculated using the following equation:

$$\text{Total}\,\text{Carbohydrate}\,\mathrm{Yield}={\text{Xylan}}_{\text{Pretreatment}}+{\mathrm{Xylan}}_{\mathrm{EnzymaticHydrolysis}}\frac{+{\mathrm{Glucan}}_{\text{Pretreatment}}+{\mathrm{Glucan}}_{\text{Enzymatic}\,\text{Hydrolysis}}}{{\text{Xylan}}_{\text{Total}}+{\mathrm{Glucan}}_{\text{Total}}}$$

The overall feedstock total carbohydrate yield thus accounts for the release of both hexose and pentose sugars from the corn stover and is used as a basis of comparison between the two pretreatment processes and impact on enzymatic hydrolysis processes between the ensiled and non-ensiled corn stover.

### Statistical Analyses

All statistical analyses were performed in the open-source language R (R Development Core Team, 2020). Important statistical tests were performed using analysis of variance (ANOVA) and statistical equivalency tests were performed using the Student’s *t*-test for two-level comparisons, or the Tukey Honest Significant Difference (HSD) test for multiple-level comparisons. All significance tests were at *p* = 0.05.

### Stereomicroscopy

Whole chopped pieces of various tissue fractions of frozen, ensiled, or pretreated corn stover were examined without further processing. Images were captured on a Nikon SMZ1500 stereomicroscope with a digital camera.

### Sample Preparation for Optical and Electron Microscopy

Three-millimeter samples of ensiled corn stover stalk rind tissue were fixed and embedded using microwave processing. Samples were fixed 2 × 6 min (2 on, 2 off, 2 on) in 2.5% glutaraldehyde buffered in 0.1 M sodium cacodylate buffer pH 7.2 (from EMS in Hatfield, Pennsylvania) under vacuum. The samples were dehydrated by treating with increasing concentrations of ethanol for 1 min at each dilution (30%, 60%, 90%, and 3 × 100% ethanol). After dehydration, the samples were infiltrated with LR White resin (from EMS in Hatfield, Pennsylvania) for 3 min with one step at room temperature (RT) overnight in increasing concentrations of resin (10%, 30%, 60%, 90%, 3 × 100% resin, diluted in ethanol). Infiltrated samples were transferred to flat-bottomed TAAB capsules and polymerized in a nitrogen-purged vacuum oven at 60°C for 24 h. LR White-embedded samples were sectioned on a Leica EM UTC ultramicrotome (from Leica in Wetzlar, Germany) with a DiATOME diamond knife.

### Immunolabeling

Sections of embedded corn stover rind were placed on ProbeOn Plus (from Fisher Scientific, Pittsburgh, PA, United States) microscope slides and incubated in a 5% non-fat dry milk w/v phosphate-buffered saline (PBS), 0.1% Tween 20 (milk/PBST)-blocking solution for 30 min at 25°C. Primary probes—PentaHIS-CBM3 (40 μg/mL milk/PBST), rat α-pectin JIM5 (1:5 v/v milk/PBST dilution) (from Carbosource, Athens, GA, United States), and 4 min, 6-diamidino-2-phenylindole (DAPI, 10 μg/mL milk/PBST) (from Molecular Probes, Eugene, OR, United States)—were applied on sections for 1.5 h at 25°C and then rinsed three times with PBST. Secondary probes – α-PentaHIS: Alexa555 (against CBM3, 1:50 milk/PBST dilution) (Qiagen, Hilden, Germany) and goat α-rat IgM:Alexa488 (against JIM5, 1:200 milk/PBST dilution) (Molecular Probes, Eugene, OR, United States) were applied on sections for 1.5 h and then rinsed 3X with PBST. Sections were dried overnight at 4°C in the dark.

### Confocal Scanning Laser Microscopy (CSLM)

Images were captured using a Nikon C1 Plus microscope (from Nikon in Tokyo, Japan), equipped with the Nikon C1 confocal system and four lasers (403 nm, 561 nm, 643 nm, and Argon tunable 458/477/488/515 nm), and operated via Nikon’s EZ-C1 software. The 435–465-nm filter was also used to detect autofluorescence. Each optical section of each channel series was scanned twice using Nikon EZ-C1 Average. For all images shown, a series of optical sections was collected, and a subset of this series was used to project the images using either Nikon EZ-C1’s Volume Render, Maximum function or ImageJ’s 3D Projection, Max function. ImageJ (from the National Institutes of Health in Bethesda, MD, United States) was used to open projected images, separate and combine color channels, and adjust contrast and brightness of images.

### Transmission Electron Microscopy (TEM)

LR White-embedded ultra-thin sections were collected on 0.35% Formvar-coated copper slot grids (from SPI Supplies in West Chester, PA, United States). Grids were post stained for 3 min with 2% aqueous uranyl acetate and 3 min with 1% KMnO_4_. Images were taken with a 4-megapixel Gatan UltraScan 1000 camera (from Gatan in Pleasanton, CA, United States) on a FEI Tecnai G2 20 Twin 200 kV LaB6 TEM (from FEI in Hillsboro, OR, United States) operating at 200 kV.

## Results and Discussion

### Evidence of Expected Eight-Week Ensiling Conditions With and Without Additives

The ensiling storage reactors were monitored for the duration of the study and showed no evidence of gas-seal failure on the reactor bodies and no indication of spoilage, however, this biomass was not included in the conversion study. At the end of the 8-week study, the moisture content of the ensiled materials was 0.51 g/g dry biomass. Organic acid concentrations in the Silamax-additive and standard- ensiled materials were 33.8 g/kg dry wt. and 35 g/kg dry wt., respectively. The measured pHs in the Silamax-additive and non-additive-ensiled materials taken at the end of the ensiling study were 4.17 and 3.95, respectively. The slight variation in organic acid levels and pHs in these two conditions is likely due to differences in between the native microbial consortia present and the consortia added with Silamax. However, these results suggest that both the ensiling preservation process and the effect of the additives occurred as expected.

### Total Structural Carbohydrates Decreased in the Ensiled Samples

The compositional analysis results of the ensiled and dried materials are shown in Table 1. All values are weight% on a dry-weight basis. The starch content of the frozen non-ensiled sample was determined to be 2.13%, but starch analysis was not performed on the dried sample. No starch was detected in either ensiled sample likely due to microbial consumption during the ensiling process.

**TABLE 1:** Compositional analysis of non-ensiled and ensiled corn stover using NREL Standard Laboratory Analytical Procedures (LAPs).

**Constituent (% w/w)**	**Non-ensiled**				**Ensiled w/o additives**		**Ensiled with additives**	
	**Frozen**	**Dried**	**Mean**	**SD**	**Mean**	**SD**		**SD**
Sucrose	1.0	4.9	2.9	2.4	1.8	0.2	1.8	0.2
Extractives	9.0	11.8	10.4	2.0	18.1	0.4	16.8	0.3
Glucan	37.9	37.0	37.5	0.8	35.3	0.4	36.0	0.1
Xylan	21.1	21.6	21.4	0.8	19.4	0.6	19.7	0.1
Galactan	2.4	2.5	2.5	0.5	0.9	0.1	0.9	0.1
Arabinan	3.4	3.9	3.6	0.9	2.0	0.2	2.1	0.1
Lignin	16.9	15.3	16.1	0.9	16.1	0.1	16.2	0.2
Ash	3.4	4.0	3.7	0.4	3.4	0.2	3.6	0.3
Total	97.8	100.0	98.9	2.0	96.3	0.5	96.4	0.4

*Data are presented on a dry matter% w/w basis.*

The two non-ensiled samples (stover frozen after harvest or dried at 45°C after harvest) collectively represent the non-ensiled materials. The compositional data for each of these samples is the average of two replicate analyses. The structural carbohydrate fractions for both non-ensiled corn stover materials were similar. The sucrose concentration was slightly lower in the frozen sample than in the dried fractions. Except for the sucrose measurement, the component values for both non-ensiled samples are within the precision of the wet chemical analysis methods (Megazyme, 2014). Thus, we treat both non-ensiled samples as from the same population. The average and standard deviation of the composition of these two samples (*n* = 4) are also shown in Table 1. The ensiled data represent duplicate analyses of triplicate samples, for a total of six measurements per condition.

The data in Table 1 shows large differences between the composition of the non-ensiled and ensiled materials. The total extractives (the sum of water and ethanol extractives) increased from 10% in the non-ensiled materials to 18% in the samples ensiled without an additive and to 17% in the samples ensiled with an additive. The glucan mass fraction decreased by an absolute average of 1.8% as a result of ensiling (all non-ensiled minus all ensiled samples), representing an average relative loss of 4.8% of the initial glucan content. Xylan content decreased by an absolute average of 1.8%, or an average relative loss of 8.4% of the initial xylan content, while galactan and arabinan content showed small relative reductions. Differences in the glucan, xylan, and water extractives content between the non-ensiled and ensiled materials were significant (*p* < 0.05). Total structural carbohydrates (the sum of glucan, xylan, galactan, and arabinan) decreased in the ensiled samples by an average of 6.7%, representing a relative loss of structural carbohydrates of 10.3%.

### Differences in Pretreatment and Enzymatic Hydrolysis Yields Between Ensiled and Non-ensiled Samples for Xylose, but Not for Glucose

**FIGURE 2:**
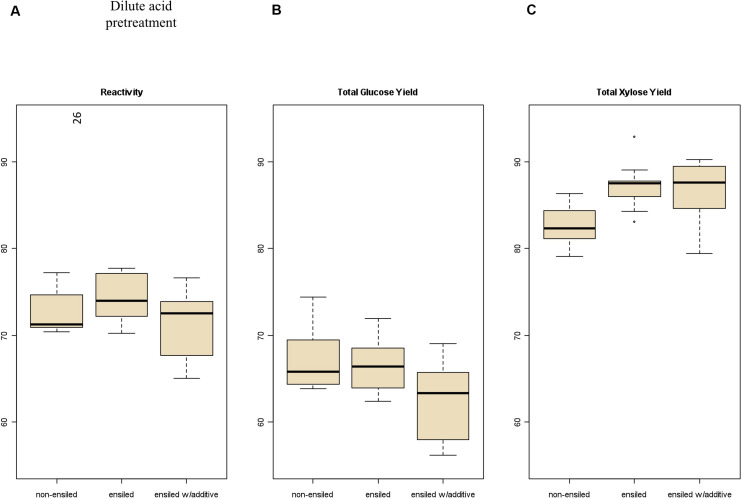
Box plots of **(A)** overall feedstock total carbohydrate yield, **(B)** total glucose yield, and **(C)** total xylose yield for dilute-acid-pretreated corn stover samples both ensiled (with and without additives) and non-ensiled. No statistically significant differences in the mean overall feedstock total carbohydrate yield or total glucose yield between ensiled and non-ensiled materials were measured.

**FIGURE 3:**
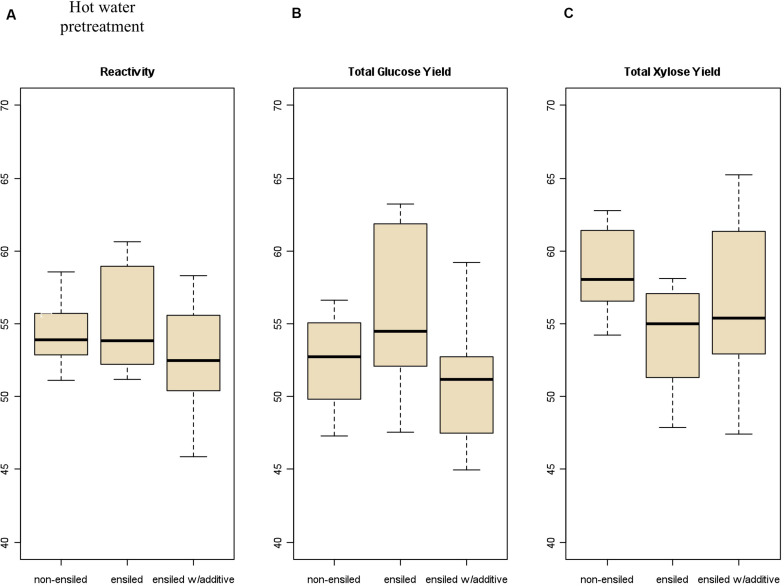
Box plots of **(A)** overall feedstock total carbohydrate yield, **(B)** total glucose yield, and **(C)** total xylose yield for hot-water-pretreated corn stover samples both ensiled (with and without additives) and non-ensiled. No statistically significant differences in overall feedstock total carbohydrate yield, total glucose yield, or total xylose yield between ensiled and non-ensiled materials were measured.

Total carbohydrate yield results from the pretreatment and enzymatic hydrolysis experiments are summarized in Figures 2, 3 for dilute-acid and hot-water pretreatments, respectively. These experiments did not reveal significant differences in overall feedstock carbohydrate yield or total glucose yield between the non-ensiled corn stover and either set of ensiled samples for either dilute-acid or hot-water pretreatment. The ensiled samples showed small but statistically significant increases in xylose yield after dilute-acid pretreatment and enzymatic hydrolysis compared to the non-ensiled samples, suggesting that the ensiling process used in this study modestly improved hemicellulose conversion. However, because the overall feedstock total carbohydrate yield calculation weights the glucose yield more heavily, these small differences did not translate to changes in overall feedstock reactivity. No differences in xylose yield were seen for the hot-water-pretreated samples.

Severe pretreatment conditions can lead to degradation of the primary sugars to unwanted byproducts (furfural from xylose degradation, HMF from glucose degradation). The samples subjected to hot-water pretreatment showed significantly higher production of HMF and furfural compared to the dilute-acid-pretreatment samples due to the longer residence time and higher temperature required for the hot-water pretreatment. Furfural concentration was higher in the hot-water-pretreated samples an average of 4.95 g/L for frozen and dried samples compared to the ensiled materials, which averaged 7.85 g/L. Furfural concentration in the acid-pretreated materials averaged 1.50 g/L for the frozen and dried samples, compared to 1.69 g/L in the ensiled materials.

**FIGURE 4:**
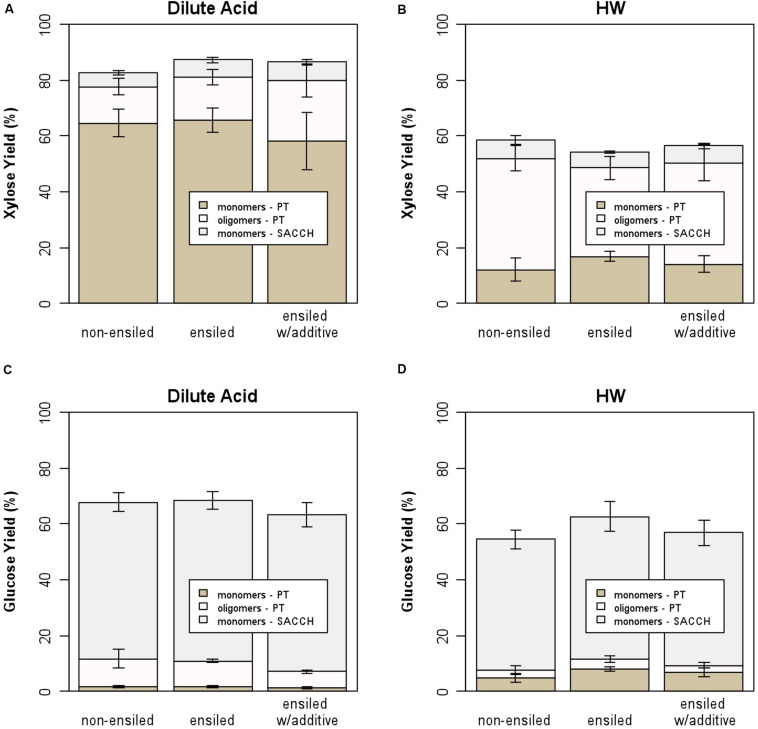
Stacked bar plot showing contribution of oligomeric and monomeric xylose and glucose for hot-water and dilute-acid pretreatment and by saccharification of the pretreated solids; **(A)** xylose yield from dilute-acid pretreatment, **(B)** xylose yield from hot-water pretreatment, **(C)** glucose yield from dilute-acid pretreatment, and **(D)** glucose yield from hot-water pretreatment. The majority of xylose is released during pretreatment, while most glucose is released from saccharification.

Figure 4 provides another way to examine the results for this work, showing the xylose and glucose yields for both dilute-acid and hot-water pretreatment followed by saccharification. For both the non-ensiled and ensiled samples, the majority of glucose was released during enzymatic hydrolysis of the pretreated solids rather than during pretreatment, while xylose release (both oligomeric and monomeric) occurred primarily during pretreatment. Hot-water pretreatment resulted in the release of much higher levels of oligomeric rather than monomeric xylose, which is consistent with previous experiments (Mosier et al., 2005). As before, there are significant differences between the ensiled and non-ensiled samples for xylose yield, but not for glucose yield. Pretreating ensiled materials at process-relevant conditions may impact sugar yields and incur additional costs due to the entrained water potentially affecting catalyst impregnation and heating requirements from the additional water. However, the water that enters with ensiled materials can reduce the water footprint required at the biorefinery and could have positive sustainability impacts. This relationship is out of the scope of this study but has been described elsewhere (Wendt et al., 2018b).

### Removal or Re-localization of Cellulose or Pectin by Ensiling Was Not Detected by Immunocytochemistry

**FIGURE 5:**
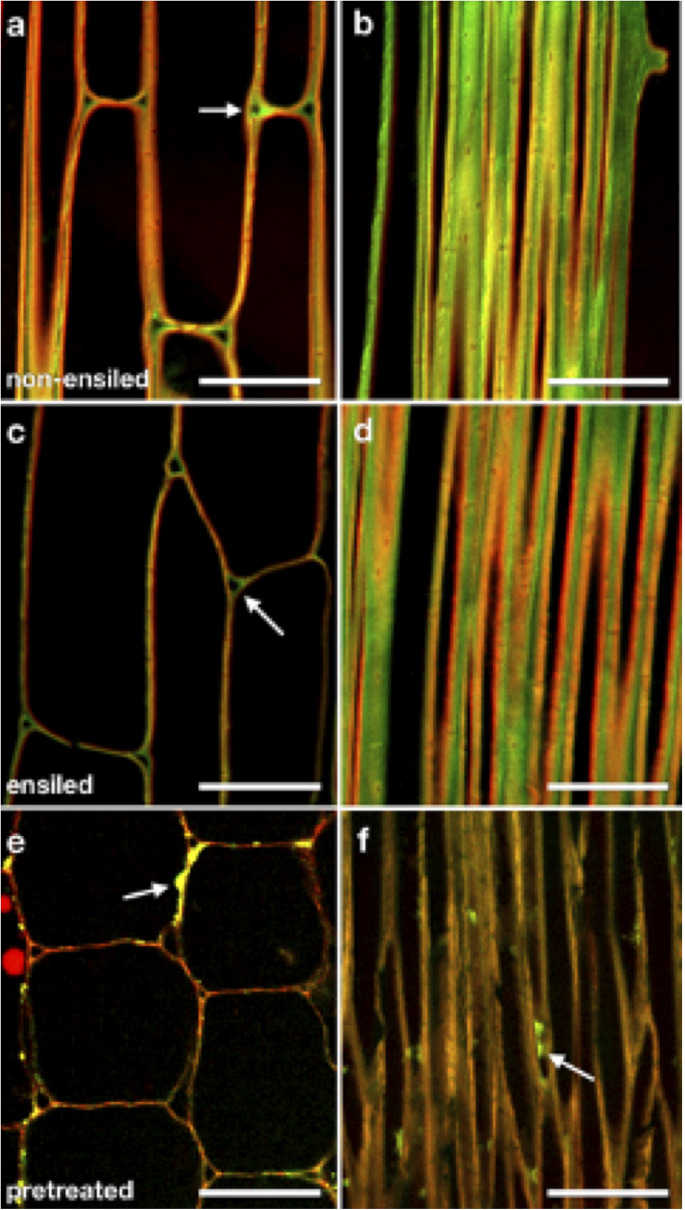
CSLM light micrographs of non-ensiled **(a,b)**, ensiled **(c,d)**, and dilute-acid pretreated **(e,f)** corn stover cell walls. Panels **(a,c,e)** are oblique transverse sections through corn stalk rind parenchyma cells. These cells have mature primary cell walls. CBM3:Alexa555 (red) and JIM5:Alexa488 (green) were used to probe cellulose and pectin accessibility, respectively. Note that when using identical probe concentrations, ensiled stover displays a cleaner, more evenly distributed fluorescent signal with clearer cellulose (red)/pectin (green) signal differentiation at the cell corners [**(a,c)** arrows]. Panels **(b,d,f)** are oblique longitudinal sections through corn stalk rind sclerenchyma cells. These cells have mature primary and secondary cell walls. Again, a pattern of less, but more discretely localized pectin is seen in the ensiled sample. The dominant features of the pretreated cell walls are a stronger cellulose-only signal, minimal pectin signal, and coalesced/redistributed cell wall material adhered to the cell wall surfaces [**(e,f)**, arrows]. Scale bars = 50 μm.

To examine whether ensiling caused any redistribution of cell wall components or changes in cell wall structure, we utilized labeling of cellulose and pectic polysaccharides with a carbohydrate-binding module (CBM) probe and specific antibodies in plastic-embedded sections of ensiled and non-ensiled corn stover rind. These probes were visualized by fluorescence CSLM. Figure 5 shows representative CSLM light micrographs of non-ensiled (a–b), ensiled (c–d), and dilute-acid-pretreated (e–f) corn stover cell walls. The images were collected from samples of the same cell and tissue types to allow comparison. Figures 5A,C,E are oblique transverse sections through corn stalk rind parenchyma cells. These cells have mature primary cell walls. CBM3:Alexa555 (red) and JIM5:Alexa488 (green) were used to probe cellulose and pectin accessibility, respectively. With these cell wall component probes, the ensiled tissue displays a cleaner, more evenly distributed fluorescent signal with clearer cellulose/pectin signal differentiation at the cell corners. Figures 5B,D,F are of oblique longitudinal sections through corn stalk rind sclerenchyma cells. These cells have mature primary and secondary cell walls. With this cell type, a similar pattern of less, but more discretely localized pectin is seen in the ensiled sample. The dominant features of the pretreated cell walls are a stronger cellulose-only signal, minimal pectin signal, and coalesced/redistributed cell wall material adhered to the cell wall surfaces (Figures 5E,F arrows).

Overall, the images of both ensiled corn stover parenchyma and fiber cells appear similar to those same cell types in non-ensiled corn stover (Figure 5). Ensiled parenchyma images show cell walls of uniform thickness compared with those of neighboring cells, as well as distinct pectin-rich middle lamellae and cell corners. Ensiled sclerenchyma images show cell walls of regular thickness as well as distinct pectic-rich middle lamellae and cellulose-rich secondary walls. Cells of ensiled stover rind, regardless of cell type, do not appear to be collapsed, nor do cell wall layers appear to be delaminated at the resolution of optical microscopy.

### Ensiled Cell Walls Exhibit Ultrastructural Characteristics Intermediate Between Non-ensiled and Dilute-Acid-Pretreated Cell Walls

**FIGURE 6:**
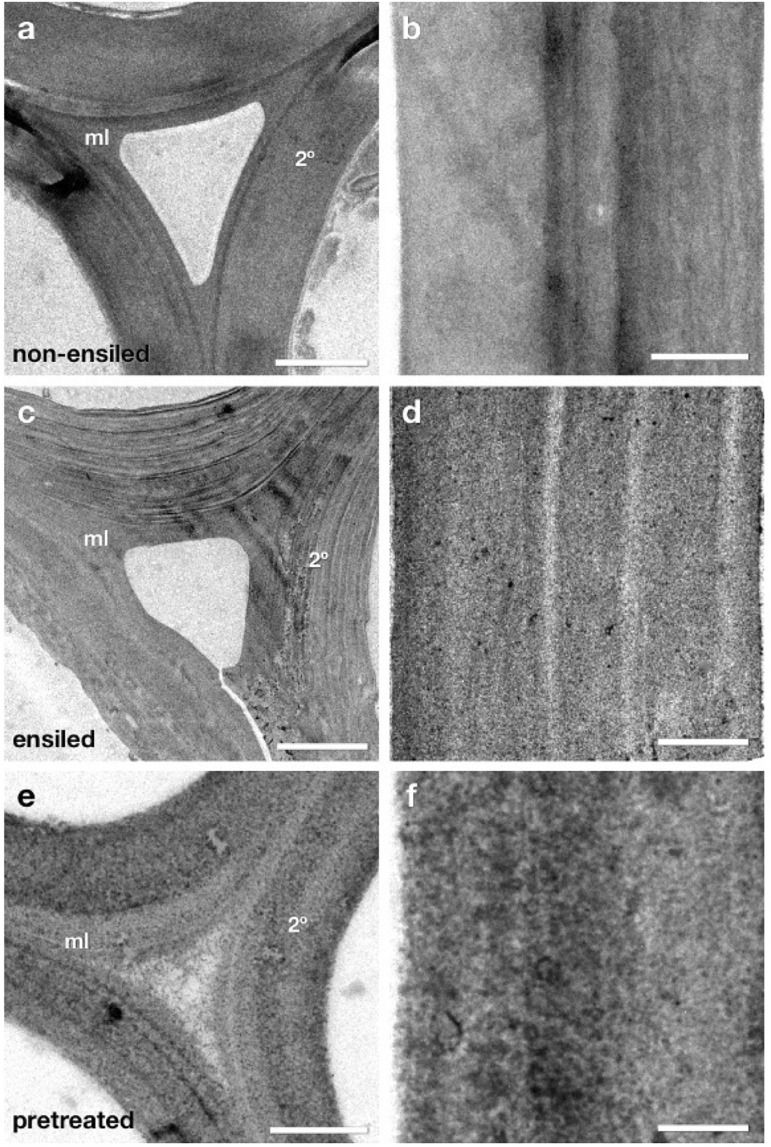
TEM micrographs of fiber cell walls from near corn stalk rind vascular bundles. These cells have mature lignified compound middle lamella (ml) and secondary (2°) cell walls. Micrographs **(a,c,e)** display a cell corner region where the curvature of the wall is greatest and the middle lamella (ml) contact between adjacent cells can be seen. Panels **(b,d,f)** show a more magnified view of secondary cell walls from each condition where the texture of the cell wall provides evidence of cell wall matrix removal and re-localization. The ensiled cell walls display some structural differences compared to non-ensiled controls. The individual lamella within the secondary cell wall are more distinct **(c)** and the staining pattern within the secondary cell wall **(d)** more granular in appearance, suggesting some removal or re-localization of cell wall components. However, as expected, the extent of removal and re-localization in ensiled walls is not as extensive as in dilute acid pretreatment **(e,f)**. Scale bars: **(a,c,e)** = 2 μm; **(b,d,f)** = 0.5 μm.

At the light microscopy level ensiled corn stover exhibited little structural change or relocation of cell wall components as detected by immunocytochemistry. However, ultra-thin sections of the same samples by TEM were analyzed to determine if more subtle structural changes were present at the ultrastructural level (Figure 6). At first glance, the ensiled cell walls again did not appear dramatically different from non-ensiled samples. There was no dramatic loss of cell wall integrity leading to collapse of cell lumen or extensive cell wall delamination, nor was there evidence of a severely degraded cell wall as seen in previous work (Donohoe et al., 2009). A more careful analysis at higher magnification, however, did reveal some ultrastructural changes that could have an impact on the further pretreatment and saccharification of ensiled biomass.

Figure 6 displays TEM micrographs of fiber cell walls from near corn stalk rind vascular bundles. These cells have mature lignified compound middle lamella (ml) and secondary (2°) cell walls. Micrographs a, c, and e display a cell corner region. Panels b, d, and f how a more magnified view of secondary cell walls from each condition where the texture of the cell wall provides evidence of cell wall matrix removal and re-localization. The ensiled cell walls display some structural differences compared to non-ensiled controls. The individual lamella within the secondary cell wall are more distinct and the staining pattern within the secondary cell wall is more granular in appearance suggesting some removal or re-localization of cell wall components. However, as expected, the extent of removal and re-localization in ensiled walls is not as extensive as in dilute acid pretreatment.

The appearance of the fine lamellar structure of the secondary cell wall in ensiled compared to non-ensiled walls implies that some of the inter lamellar connections have weakened or been partially removed (Figure 6). Similar lamellar separation patterns enhanced by pretreatments have been observed in previous studies (Donohoe et al., 2009). At higher resolution the subtle differences among non-ensiled, ensiled and pretreated cell walls are revealed. The fine pattern of distribution of low and high densely staining material in the non-ensiled sample is typical of the natural density and distribution of the hemicellulose and lignin in native cell walls. In a dilute acid pretreated sample, the pattern of dense material has become coarser. In previous work, we have shown that this pattern is partly due to the extraction of hemicellulose and the coalescence and migration of lignin within the cell wall (Donohoe et al., 2008). While ensiled cell walls do not exhibit the same coalescence pattern as the dilute acid pretreated material, they do have a different structure and coarser staining pattern than the control. This pattern is consistent with some extraction and reorganization of the cell wall matrix components and a partial loosening of the wall structure evidenced by a lower overall electron density and regions of visibly increased porosity. These ultrastructural observations also suggest that alternative conversion methods, such as deacetylation and mechanical refining (DMR), that could take advantage of the lamellar defects and may be more effective than dilute acid or hot water pretreatment for biomass conversion of ensiled materials.

## Conclusion

Providing year-round feedstock supply having consistent quality, quantity, cost, and stability is a major challenge to future biorefineries. Traditional bale systems, while representing the current preferred method for storage of agricultural residues, are limited due to dry-matter losses, requirements for in-field drying, and fire risk. Wet storage by ensiling agricultural residues offers the potential for reducing these risks. The high moisture content coupled with an anaerobic environment, at low pH reduces material losses providing more options for collection and storage. Ensiling and ensiling with additives did not reduce the bioconversion requirements of the carbohydrates from either dilute acid or hot water pretreatment of corn stover. Additional technoeconomic analysis is necessary to determine if the cost/benefit of ensiling offsets the loss of biomass carbohydrates and the additional transportation cost from the entrained water associated with wet storage.

## Data Availability Statement

The raw data supporting the conclusions of this article will be made available by the authors, without undue reservation, to any qualified researcher.

## Author Contributions

NN and EW planned the experiments, analyzed the conversion results, and wrote the manuscript. EW conducted statistical analysis of the conversion results. BD designed the microscopy and image analysis and wrote the manuscript. NW and EK conducted pretreatment and enzymatic hydrolysis experiments used in this study and contributed to the manuscript. TH performed the microscopy and image analysis. All authors read, edited, and approved the final draft of the manuscript.

## Conflict of Interest

CR was employed by the company Verd Company. The remaining authors declare that the research was conducted in the absence of any commercial or financial relationships that could be construed as a potential conflict of interest.
